# EDITORIAL COMMENT: LACK OF EVIDENCE OF HPV ETIOLOGY OF PROSTATE CANCER FOLLOWING RADICAL SURGERY AND HIGHER FREQUENCY OF THE ARG/PRO GENOTYPE IN TURKISH MEN WITH PROSTATE CANCER

**DOI:** 10.1590/S1677-5538.IBJU.2015.0429.1

**Published:** 2017

**Authors:** Jose Pontes

**Affiliations:** 1 Departamento de Urologia Hospital das Clincas da FMUSP, Instituto Central São Paulo, SP, Brasil

The authors retrospectıvely evaluated the presence of HPV and the p53 polymorphism distribution by RT-PCR in a series of 60 surgical specimens of localized prostate cancer and 36 BPH samples. They found HPV in only one prostate cancer case and no HPV in BPH cases. They also demonstrated that Ar72Pro polymorphism and the Proline allele were more frequently found in cancer. They concluded that there is no association between prostate cancer and HPV and also postulate that the Proline allele can be a risk factor for the development of PCa in the Turkish population.

The article’s subject is not original, the author (1) themselves recognized that there is a plenty of data about the association of HPV infection and prostate cancer risk in literature. The novelty relies in the correlation between the p53 polymorphisms and the prostate cancer prognostic factors Gleason score, pathologic stage, and ISUP grading. The small sample size is a serious limitation of this paper, specialy to infer about genotype frequences in a specific population, which the authors have already recognized at discussion section. While not original and statisticaly underpowered I think that article brought some data that expanded our knowledge of prostate carcinogenesis.
